# Genetic and molecular insights into tiller development and approaches for crop yield improvement

**DOI:** 10.3389/fpls.2025.1532180

**Published:** 2025-03-27

**Authors:** Zaid Chachar, Xiaoming Xue, Junteng Fang, Ming Chen, Weiwei Chen, Xuhui Li, Nazir Ahmed, Sadaruddin Chachar, Aamir Ali, Zhong liang Chen, Lina Fan, Ruiqiang Lai, Yongwen Qi

**Affiliations:** ^1^ College of Agriculture and Biology, Zhongkai University of Agriculture and Engineering, Guangzhou, China; ^2^ Institute of Nanfan and Seed Industry, Guangdong Academy of Science, Guangzhou, Guangdong, China; ^3^ College of Horticulture and Landscape Architecture, Zhongkai University of Agriculture and Engineering, Guangzhou, China; ^4^ College of Agriculture, Shanxi Agricultural University, Jinzhong, Shanxi, China; ^5^ Key Laboratory of Sugarcane Biotechnology and Genetic Improvement (Guangxi), Ministry of Agriculture and Rural Affairs/Guangxi and Key Laboratory of Sugarcane Genetic Improvement/Sugarcane Research Institute, Guangxi Academy of Agricultural Sciences, Nanning, China

**Keywords:** tiller development, quantitative trait loci (QTL), genome-wide association studies (GWAS), transcriptome analysis, genes

## Abstract

Tiller development is a critical factor in boosting agricultural productivity and securing global food security. This review offers a comprehensive analysis of recent advancements in enhancing crop yield through extensive research on tiller development, utilizing a multi-faceted approach that includes quantitative trait loci (QTL) mapping, association studies, and transcriptome analysis across various crops. Extensive investigations have revealed complex genetic, molecular, and environmental interactions that influence this pivotal yield determinant. QTL mapping has pinpointed specific genomic regions associated with tiller development, while genome-wide association studies (GWAS) have provided deeper insights into natural genetic variations within populations. Additionally, transcriptome analyses have offered a dynamic view of gene expression, shedding light on molecular regulatory mechanisms that govern tillering. The integration of these multi-omics approaches has enabled a holistic understanding of the process, identifying crucial genetic loci and expression patterns that are key to optimizing tillering. Key genes such as *TaMAX1, TaMOC1*, and *TN1 in wheat, ZmTB1, ZmD14, and ZmMOC1* in maize, along with *MAX1*-like genes, *OsMAX1, and OsHAM2* in rice have been highlighted. Similar studies in sugarcane have identified genes like *SoMAX2, SoMAX3*, *SoMAX4-1, SoMAX4-2*, and *SoTB1*, which regulate bud outgrowth and tillering. Including involving hormonal control integrates pathway auxins, gibberellins, and cytokinins, to coordinate plant responses to internal and external stimuli “These” discoveries are essential for breeding and genetic engineering strategies aimed at developing crop varieties with favorable tillering traits, ultimately enhancing yield potential.

## 1 Introduction

Tiller development in cereal crops, such as wheat, rice, barley, and maize, is critical for boosting agricultural productivity and ensuring global food security (Grote et al., 2021). Tillers are lateral shoots emerging from the plant base, and their formation directly impacts the reproductive success of these staple cereals (Luna et al., 2016). A higher number of healthy tillers leads to optimal stalk density, essential for achieving high yields. Factors influencing tillering include crop variety, light exposure, temperature, soil moisture, planting density, and fertilization practices. Light plays a particularly pivotal role, as sufficient light at the plant base stimulates vegetative buds, thereby promoting tiller formation (Fernando Santos, 2015). In recent years, climate change has intensified challenges in managing tiller development. Extreme weather events like prolonged droughts and heatwaves disrupt tiller initiation and reduce tiller viability. High temperatures during critical growth phases can alter hormonal balances necessary for bud outgrowth, leading to fewer tillers and reduced yield potential (Karavolias et al., 2021). Drought further exacerbates these effects by limiting water availability, essential for nutrient uptake and cell division, which curtails tillering, especially in moisture-sensitive crops like wheat and rice. These climate-driven changes underscore the urgency of developing resilient crop varieties capable of sustaining tiller production under stress.

Climate change affects tillering by altering environmental factors such as temperature, water availability, and atmospheric CO_2_ levels, all of which directly impact plant physiology and gene expression. Temperature stress, for example, disrupts hormonal pathways that regulate axillary bud outgrowth, reducing tiller numbers. In rice (*Oryza sativa*), high temperatures accelerate senescence in young tillers and disrupt auxin distribution, leading to reduced tillering. The *MONOCULM 1*(*MOC1*) gene, which promotes tiller formation, is downregulated under high temperatures, limiting tiller production (Hussien et al., 2014). In wheat (*Triticum aestivum*), temperature stress affects *SPL* genes like *TaSPL14*, critical regulators of tillering, with reduced expression further limiting tiller formation and impacting grain yield (Kebrom et al., 2012).

Drought stress, another climate change impact, reduces tillering by affecting cytokinin synthesis and disrupting hormonal balances in axillary buds. In maize (*Zea mays*), drought conditions inhibit tiller formation by altering the expression of genes such as *ZmD14*, which interacts with cytokinin and strigolactone pathways, prioritizing survival over branching (Fan et al., 2023). Similarly, in barley (*Hordeum vulgare*), the drought-responsive *HvSPL14* gene is downregulated, leading to a reduction in tiller numbers and resilience under prolonged dry spells (Valivand, 2023). Elevated CO_2_ levels present a more complex influence on tillering. While increased CO_2_ can enhance photosynthesis and stimulate tillering due to improved carbohydrate availability, prolonged exposure often causes plants to shift resources to the main stem, reducing final tiller numbers. This effect has been linked to altered expression of genes like *OsSPL14* in rice, where elevated CO_2_ levels shift hormonal balances in favor of primary growth over branching (Wang et al., 2015). These challenges highlight the need for climate-resilient crop varieties with robust tillering traits. Molecular breeding offers promising solutions by targeting genes that confer tolerance to environmental stresses, such as temperature-resistant *TaSPL14* variants in wheat or drought-tolerant *HvSPL14* in barley. Strengthening tiller development is crucial for stabilizing yields amidst increasingly variable climate conditions.

Agronomically, the number and health of tillers correlate directly with grain yield, as each tiller has the potential to produce a panicle or inflorescence, contributing to total grain production (Bauer and von Wirén, 2020). Tillering is thus a vital growth characteristic with significant implications for agricultural practices and food security. A plant’s ability to maintain healthy tillers under variable conditions is a measure of its adaptability and resilience traits that are increasingly valuable in the face of climate change and unpredictable weather patterns (Yu et al., 2019). Environmental stresses, including drought and nutrient deficiencies, adversely affect tiller viability, ultimately reducing crop yields (Karavolias et al., 2021). Consequently, enhancing tiller development is a priority in crop improvement programs, with a focus on sustainable agricultural practices.

This review synthesizes research on tiller development through quantitative trait loci (QTL) mapping, association studies, and transcriptome analysis across diverse crops. Despite progress, an integrated understanding that combines these multi-omic approaches to decipher the regulation of tiller development remains limited. This review aims to bridge existing knowledge gaps by examining genetic loci, molecular pathways, and environmental factors that influence tillering in cereals. By consolidating current findings and identifying areas for further research, this paper provides insights for crop breeding and genetic engineering to develop high-yield cereal varieties optimized for tillering. A key aspect of tiller development is genetic control involving genes such as *TaSPL14* in wheat, *ZmTB1* in maize, and *OsMAX1* in rice, each regulating tiller numbers. These genes are part of larger signaling pathways influenced by phytohormones like strigolactones, auxins, and cytokinins, essential for plant branching and bud outgrowth. Strigolactone biosynthesis, which regulates plant architecture, involves genes across various species. For instance, *TaMAX1* in wheat, *OsMAX1* in rice, and *SoMAX* genes in sugarcane (e.g., *SoMAX2*, *SoMAX3*, *SoMAX4-1*, and *SoMAX4-2*) encode enzymes like cytochrome P450 (CYP711A1) and carotenoid cleavage dioxygenases, converting carotenoids to carlactone, a precursor for strigolactone compounds that limit shoot branching and promote root development under low-nutrient conditions. This paper advocates for a multidisciplinary approach to tackle the challenges climate change poses to tillering, highlighting the necessity of resilient crop varieties that can withstand environmental stresses. Through this comprehensive analysis, we aim to set a direction for future research and foster collaborative efforts to advance the understanding and manipulation of tiller development, ultimately contributing to sustainable food production.

### 1.1 Challenges in improving tiller development.

Enhancing tiller development in crops, a key determinant of agricultural yield, is a complex task due to the multifaceted interactions among genetic, environmental, and physiological factors. Tiller development is polygenic, with multiple genes interacting to regulate this trait, which complicates breeding efforts (Mackay and Powell, 2007). Climate change further intensifies these challenges. For example, elevated temperatures can accelerate plant metabolism, potentially shortening the tillering phase and reducing tiller numbers. Conversely, unexpected cold spells may inhibit tiller initiation, disrupting the seasonal timing of crop development and maturity (Qaderi et al., 2019). Soil moisture variations, caused by drought or excessive rainfall, also have a major impact on tillering. Drought conditions limit water availability, impairing nutrient uptake and leading to poor tiller growth and early senescence, which ultimately reduces yield. In contrast, waterlogged soils limit oxygen availability, stressing roots and stunting tiller formation (Karavolias et al., 2021). Addressing these climate-induced challenges is essential for developing resilient, high-yielding crop varieties adapted to changing environmental conditions. Given this complexity, a holistic approach to breeding and genetic engineering is necessary to optimize tillering traits across diverse agro-ecological zones (Zhao S. et al., 2020). The genotype-environment interaction complicates breeding for tillering improvement, as the expression of tillering traits varies significantly with environmental conditions, making it challenging to predict a genotype’s performance across settings (Qaderi et al., 2019).

For instance, in wheat, the expression of *TaMOC1*, a gene regulating tiller bud initiation, fluctuates in response to environmental triggers such as temperature and nutrient availability. Research indicates that *TaMOC1* expression increases under cooler temperatures, promoting tillering, while warmer conditions suppress its expression, ultimately affecting yield (Yang et al., 2023). This variability highlights the need for breeding programs to consider environmental adaptability in selecting key tillering genes. Similarly, in rice, *OsSPL14* expression responds to nitrogen levels, with lower nitrogen reducing its expression and, consequently, tiller numbers. Breeding programs focused on optimizing nitrogen-use efficiency must account for such gene-environment interactions to stabilize tiller production under varying soil nutrient levels. Incorporating these interactions into breeding strategies enables the development of robust cultivars suited to diverse environmental conditions.

A systematic review of tiller development across cereal crops including sugarcane, maize, wheat, and rice identifies eight primary challenges in optimizing tillering: genetic limitations, nutrient availability, water stress, light intensity and quality, temperature stress, pest and disease pressures, plant density and spacing, and balanced trait breeding. Each challenge presents unique hurdles in cultivation (Nilahyane et al., 2023). For example, genetic limitations highlight varietal constraints on tillering potential, while nutrient availability, particularly nitrogen, plays a crucial role in tiller formation. Water stress, whether due to drought or waterlogging, alongside challenges in managing light and temperature, significantly impact tillering (Cabello et al., 2023; Khedwal et al., 2023). Additionally, pest and disease pressures necessitate careful management to maintain plant health.

While these challenges are considerable, integrating genomics and transcriptomics into agricultural research offers promising solutions. Genomics, the study of the complete DNA of an organism, has expanded our understanding of the genetic basis of tiller growth (Kushwaha et al., 2022; Shang et al., 2021; Yan et al., 2023). Techniques such as Quantitative Trait Loci (QTL) mapping and Genome-Wide Association Studies (GWAS) have identified key genetic segments and markers associated with tillering traits. These markers are invaluable in guiding breeding efforts to develop crop varieties with optimized tiller numbers, essential for enhancing productivity (Li M. et al., 2022). Genomic research not only improves our understanding of the genes and pathways crucial for tillering but also finds practical application in crop breeding. By identifying specific genes, breeders and researchers can modify them using genetic engineering techniques to enhance tillering traits. This progress is vital for sustainable agriculture, as it boosts yields without increasing resource use (Hu et al., 2018). Complementing genomics, transcriptomics examines the complete set of RNA transcripts in a plant, revealing active gene expression and how genes respond to environmental conditions (Bohra et al., 2020). This approach is particularly useful for studying gene-environment interactions that influence tillering, aiding in the development of resilient, high-yielding crop varieties (Wang X. et al., 2020). With advancements in high-throughput sequencing and bioinformatics, genomic and transcriptomic research now spans a broader range of crops, deepening our understanding of tillering mechanisms across species. Together, genomics and transcriptomics provide a powerful toolkit for addressing the challenges in improving tiller development. By offering an in-depth view of the genetic and molecular foundations of tillering, these disciplines are ushering in a new era in crop breeding and genetic engineering, with the potential to sustainably revolutionize crop productivity.

## 2 Tiller development in crop plants

Tiller development in crop plants, particularly in grasses and cereal crops, is a vital component of their growth, directly influencing yield potential. This process begins morphologically with the initiation of axillary buds at the plant’s base. Whether these buds remain dormant or develop into tillers is determined by a complex interaction of genetic and environmental factors. The formation of a tiller involves the emergence of a new shoot comprising leaves, stems, and, in the case of cereal crops, a flowering head, which significantly contributes to grain yield potential (Riaz et al., 2023; Shang et al., 2021).

Genetically, tiller development is regulated by a network of genes that influence various stages of growth, from bud initiation to outgrowth and sometimes even senescence. Notable among these regulatory genes are those in the TEOSINTE BRANCHED1, CYCLOIDEA, and PCF (TCP) family, which play a central role in controlling tillering and plant architecture in key crops like rice and maize. Additionally, plant hormones are vital in modulating this developmental process. Auxins, primarily produced in the shoot apex, act to suppress axillary bud growth, thereby controlling tiller numbers. In contrast, cytokinins promote bud growth, while strigolactones are a class of plant hormones crucial for managing shoot branching serve to inhibit tiller outgrowth. These hormones collectively create a balancing mechanism for resource allocation and growth management within the plant (Shang et al., 2021; Sosnowski et al., 2023).

Environmental conditions also significantly impact tiller development. Factors such as light availability, temperature, and nutrient supply, especially nitrogen, are critical in determining the extent of tillering. Adequate light and optimal temperature conditions favor tiller growth, while nutrient status, particularly nitrogen availability, directly influences the vigor and number of tillers a plant can support (Yan et al., 2023). Additionally, tiller development is intricately linked with the plant’s overall physiological state, including the distribution of energy and nutrients among various plant parts (Gracey, 2007). This dynamic interaction ensures a balance between vegetative growth and the reproductive needs of the plant, a balance that is essential for both immediate survival and long-term reproductive success.

The biological basis of tiller development in crop plants is thus a multifaceted and dynamic process, shaped by genetic, hormonal, and environmental factors, as well as the plant’s internal physiological state. Understanding these complex interactions is essential for devising strategies to optimize tillering, thereby enhancing agricultural productivity and food security. Key genes like MOC1 in rice, TB1 in maize, and TaSPL14 in wheat illustrate the profound influence of gene expression regulation on tiller numbers and overall plant growth patterns. By manipulating the expression of these genes, through either traditional breeding or advanced genetic engineering techniques, it is possible to develop crop varieties tailored to specific growing conditions, optimizing tillering for maximum yield.

### 2.1 The negative and positive impact of tillers

In studying the multifaceted impacts of tillering on crop production, it becomes evident that tillering plays a crucial role in both enhancing and challenging agricultural outcomes. The positive impacts of tillering are substantial, particularly in terms of increased yield. The presence of additional tillers on a plant leads to the formation of more grain heads, which inherently maximizes grain production per plant and boosts overall field yields. This efficiency in space and resource utilization is particularly valuable in optimizing crop density and compensating for early-season plant losses, thereby maintaining uniform crop stands. Moreover, the adaptability conferred by tillering enhances crop resilience to environmental stresses, allowing crops to better withstand adverse conditions. This adaptability is further augmented by the improved resource use efficiency associated with tillers, which facilitates increased light interception, enhanced nutrient uptake, and better water use efficiency. The genetic diversity expressed through tillering also presents opportunities for breeding programs aimed at improving crop adaptability and productivity (Jungers et al., 2023).

On the flip side, the negative impacts of tillering underscore the complexity of managing tiller growth (**Table 1**). Resource competition between tillers and the main stem can diminish plant vigor and reduce grain size and quality, particularly under conditions of limited resource availability. The denser canopy resulting from excessive tillering can create microenvironments that favor the proliferation of pathogens, posing increased disease pressure and necessitating more intensive management strategies (Shang et al., 2021). Harvesting challenges emerge from the uneven maturity and increased biomass associated with tillers, complicating the timing and mechanics of harvest operations and potentially affecting grain quality. Understanding the dual nature of tillering’s impacts is essential for developing informed strategies that leverage its benefits while mitigating its drawbacks. This nuanced approach to tillering management promises to enhance agricultural productivity and sustainability in cereal crop production.

**Table 1:** Impacts of tillering in crop production.

Impact Type	Impact Description and Key Points	References
Positive Impacts		
Increased Yield	More tillers lead to additional grain heads, maximizing grain production per plant and enhancing overall field yields, which ensures better use of space and resources.	(Türkösi et al., 2023; Yan et al., 2023)
Compensation for Plant Loss	Tillers fill in for plants that are damaged or fail to emerge, reducing the impact of early-season losses and helping maintain uniform crop stands for optimal use of planted area.	(Haag et al., 2017)
Improved Resilience	The ability to produce tillers improves a crop’s resilience to environmental stresses by enhancing survival under adverse conditions and allowing adaptation to variable environments.	(Manenti et al., 2023; Varshney and Bohra, 2023)
Enhanced Resource Use	Tillers increase the plant’s ability to capture and utilize available resources, leading to increased light interception, enhanced nutrient uptake, and improved water use efficiency.	(Mason et al., 2020; Ullah et al., 2019)
Genetic Diversity Expression	Tillering allows the expression of genetic potential for adaptability and yield, which can be leveraged in breeding programs to improve crop resilience and productivity.	(M et al., 2023)
Negative Impacts		
Resource Competition	Tillers compete with the main stem for critical resources, potentially reducing growth and vigor and leading to smaller, less filled grain heads due to nutrient dilution.	(Alzueta et al., 2012; Wang et al., 2017)
Increased Disease Pressure	A denser canopy from excessive tillering can favor the development and spread of pathogens, requiring more intensive disease management strategies.	(Lestari, 2015; Mawar and Lodha, 2022)
Harvesting Challenges	Uneven maturity and increased biomass from tillers can delay harvest and increase the difficulty and cost of mechanical harvesting, potentially reducing grain quality.	(Duganets et al., 2023)
Altered Crop Microclimate	The dense canopy created by tillers can alter the crop’s microclimate, affecting pollination and grain filling, and may increase moisture retention, promoting disease.	(Karthika et al., 2022)

### 2.2 Genetic basis of tiller development

#### 2.2.1 Functions of key regulatory genes and plant architecture

The genetic regulation of tiller development in crops is controlled by key regulatory genes that shape plant architecture through complex networks involving hormonal signals, environmental cues, and genetic interactions. A central element in this regulatory network is the strigolactone pathway, with genes such as *TaMAX1* in wheat (*Triticum aestivum*) an ortholog of the *Arabidopsis thaliana* MAX1 gene playing a critical role in strigolactone biosynthesis. These networks integrate strigolactone, auxin, and cytokinin signaling to regulate tiller bud dormancy and outgrowth, balancing growth based on environmental resource availability.

The core mechanisms governing tillering appear to be highly conserved across cereal crops, with key genes such as *TB1*, *MOC1*, *MAX1*, and *SPL14* functioning similarly in wheat, maize, rice, and sugarcane. These genes participate in hormone signaling pathways, particularly strigolactone and cytokinin regulation, which influence axillary bud outgrowth. However, comparative studies indicate that while the genetic framework remains largely conserved, species-specific variations exist in gene expression patterns and functional adaptations.

Beyond cereals, legumes and vegetable crops exhibit distinct yet partially overlapping regulatory mechanisms for shoot branching. For instance, in legumes such as soybean (*Glycine max*) and pea (*Pisum sativum*), the *Rms* (Ramosus) and *Dt* (Determinate) genes regulate branching through strigolactone and auxin pathways, resembling the functions of *TB1* and *MAX* genes in cereals. Similarly, in tomato (*Solanum lycopersicum*), *Ls* (Lateral suppressor) and *Blind* genes contribute to lateral shoot development through auxin-mediated suppression, showing functional parallels with *MOC1* in rice.

These findings suggest that while core genetic regulators of tillering and branching are conserved across monocots and dicots, variations in their expression and interaction with hormonal networks allow species to adapt their shoot architecture to different ecological and agronomic needs. Future research integrating comparative genomics across cereals, legumes, and vegetables could provide valuable insights into the evolution and diversification of these regulatory pathways, aiding in targeted breeding strategies for multiple crop types.

Strigolactones act as suppressors of tiller growth, limiting axillary bud outgrowth under nutrient-limited conditions through genes like *D14*, *MAX1*, and *D3* in rice, maize, and wheat. In particular, *TaMAX1* in wheat and *OsMAX1* in rice catalyze key biosynthetic steps, converting precursor molecules into active strigolactones (Gupta et al., 2023). This process is complemented by auxin and cytokinin, where auxin from the shoot apex enforces apical dominance by suppressing lateral bud growth, as seen with the *TB1* gene in maize. In contrast, cytokinins promote bud outgrowth, with genes like *OsCKX* in rice reducing tillering under limited resources by accelerating cytokinin degradation (Doebley et al., 1997). Transcription factors, such as SPL (SQUAMOSA PROMOTER BINDING PROTEIN-LIKE) genes, integrate hormonal cues to modulate tiller formation. For instance, *OsSPL14* in rice and its wheat ortholog *TaSPL14*, regulated by miR156, are linked to reduced tillering and robust stem growth, optimizing yield (Wang et al., 2015). Together, these genes create a regulatory network that adapts tiller development to environmental changes, crucial for breeding high-yield, resilient crops.

In wheat, functional variants like *TaMAX1a2-3B* have been identified. For instance, *TaMAX1a2-3B* from *Triticum dicoccoides*, encoding a 540-amino-acid protein, is confirmed as a functional MAX1 homolog (Sigalas et al., 2023). Gene-environment interactions also affect gene expression; for example, *TaMAX1* expression increases in low-phosphate conditions, producing more strigolactones, which in turn suppresses tiller outgrowth, conserving resources for root development (Cheng et al., 2013). Similarly, drought conditions upregulate *ZmD14* in maize, reducing lateral shoot growth to conserve water (Fan et al., 2023).

The *TaMAX1* gene, encoding a cytochrome P450 enzyme (CYP711A1), catalyzes the conversion of carlactone into bioactive strigolactones, critical for limiting shoot branching and enhancing root growth. This pathway promotes nutrient uptake and facilitates symbiosis with arbuscular mycorrhizal fungi, which further aids in soil nutrient absorption (Cheng et al., 2013; Faizan et al., 2020). The interaction of strigolactones with auxins, gibberellins, and cytokinins provides a nuanced balance between shoot and root growth in response to environmental signals, optimizing plant architecture for resource efficiency and resilience. TaMOC1 in wheat is an ortholog of the rice MOC1 gene, a pivotal regulator of tiller bud outgrowth. It plays a critical role in initiating and developing tiller buds, promoting the formation of lateral shoots and potentially increasing crop bushiness and grain yield. As a GRAS family transcription factor, TaMOC1 influences other genes involved in meristem initiation and responds to environmental and internal growth signals. This makes TaMOC1 a key target for genetic modifications aimed at optimizing wheat’s tillering patterns and overall architecture. TaMOC1 regulates tillering through a complex gene regulatory network that integrates environmental signals and hormonal controls, particularly interactions with cytokinins and strigolactones, which stimulate and suppress tiller growth, respectively (Yang et al., 2023; Zhang et al., 2015).

ZmTB1 in maize is an ortholog of Arabidopsis’s TB1, regulating plant architecture by controlling tillering and branching. It suppresses lateral branching, contributing to the single-stalk growth habit typical of modern maize. This trait differentiation has been instrumental in maize’s domestication and breeding. ZmTB1 modulates the plant’s hormonal balance, particularly affecting gibberellins and cytokinins, which influence growth and branching. This gene typically reduces sensitivity to gibberellins in bud cells, promoting vertical growth over branching (Mansilla et al., 2023; Yan et al., 2020; Fan et al., 2023).

ZmD14 in maize plays a critical role in strigolactone signaling by functioning as a receptor for these hormones, which are crucial in suppressing shoot branching and altering plant growth patterns. When strigolactones bind to ZmD14, it triggers a signaling cascade that results in the suppression of lateral shoot development (Mansilla et al., 2023; Fan et al., 2023). ZmMOC1 in maize influences the formation and outgrowth of tillers, mirroring its ortholog’s function in rice. It likely encodes a GRAS family transcription factor that directly influences the transcriptional regulation of genes essential for meristem development and activity. ZmMOC1 controls tiller bud outgrowth by modulating the expression of downstream genes, integrating growth factors and hormonal signals within the plant (Yan et al., 2020).

In rice, OsMAX1 plays a central role in the biosynthesis of strigolactones, critical hormones that regulate plant architecture by controlling shoot branching. This gene’s product, a cytochrome P450 enzyme, facilitates the conversion of carlactone into bioactive strigolactones, optimizing plant structure for better energy efficiency.

In sugarcane, genes such as SoMAX2, SoMAX3, SoMAX4-1, and SoMAX4-2 are involved in the strigolactone signaling pathway, which regulates plant architecture by controlling the biosynthesis of strigolactones. These genes likely influence shoot branching, promoting a more productive plant structure. They encode enzymes essential for converting carotenoids into strigolactones, which regulate growth by suppressing excessive shoot branching and optimizing resource allocation. The strigolactone pathway also interacts with other hormonal pathways, such as those controlled by auxins and cytokinins, to balance plant growth with environmental cues (Karno, 2007).

SoTB1 in sugarcane, a homolog of maize’s TB1, likely plays a similar role in regulating tillering. It suppresses the development of lateral branches to promote more robust main stalks. SoTB1 interacts with several hormonal pathways, notably gibberellins and cytokinins, which generally promote shoot branching. SoTB1 modifies the plant’s response to these hormones, possibly reducing the sensitivity of axillary buds to cytokinin-induced growth signals, thereby maintaining them in a dormant state. Additionally, SoTB1 may interact with the strigolactone pathway, where strigolactones strengthen the inhibitory effect on shoot branching, effectively integrating multiple hormonal signals to control plant architecture. Understanding these molecular mechanisms and regulatory pathways provides valuable insights into crop management and improvement strategies. This knowledge is pivotal for developing strategies to enhance crop yield and adaptability by optimizing nutrient use and adjusting plant growth patterns to suit various agricultural needs (Karno, 2007).

**Figure 1:**
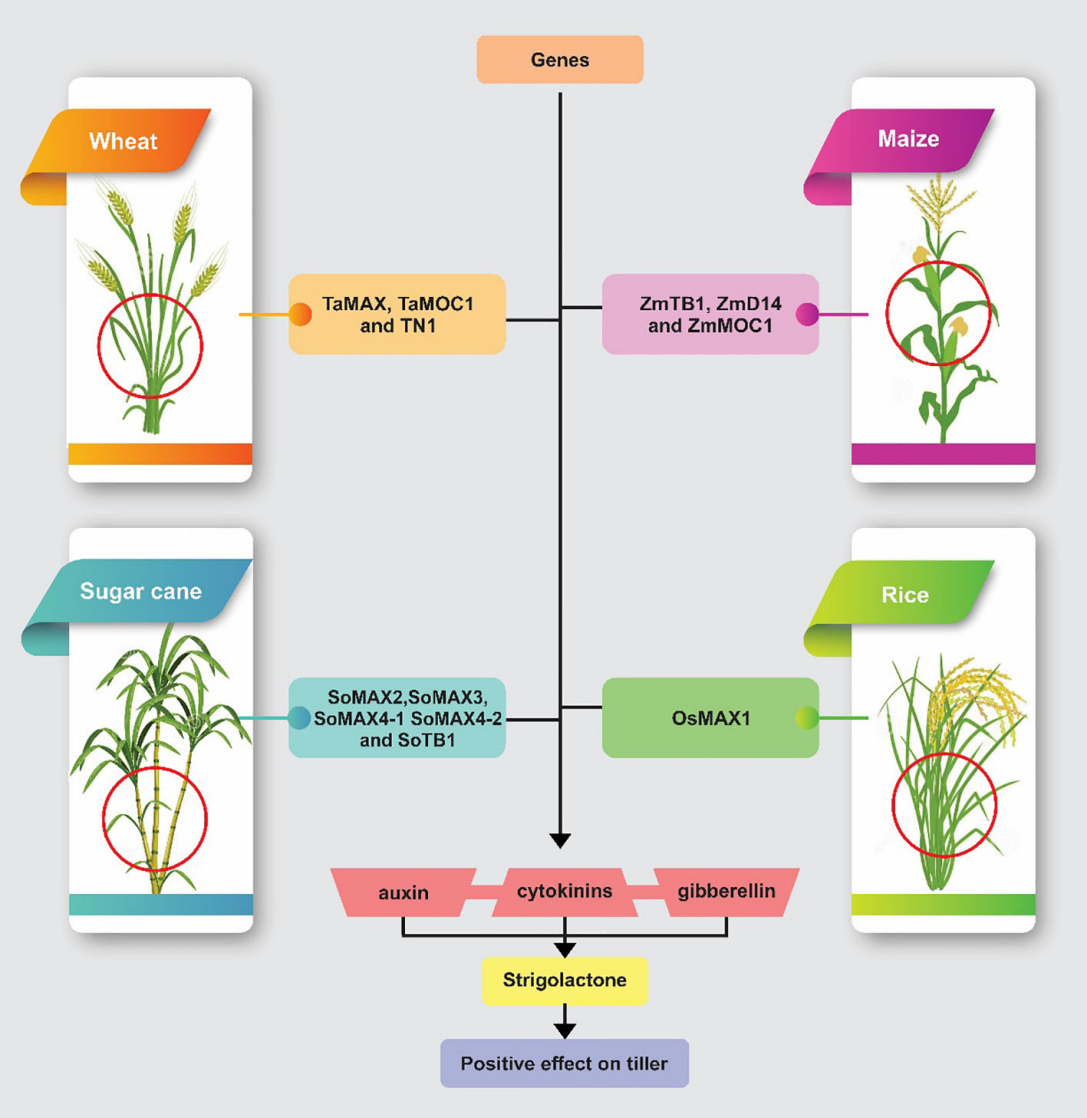
This figure illustrates how specific genetic factors in wheat, maize, rice, and sugarcane contribute to plant architecture and tillering through the modulation of hormonal pathways. In wheat, genes such as TaMAX1, TaMOC1, and TN1 influence tiller development through interactions with auxins, gibberellins, and cytokinins. In maize, ZmTB1, ZmD14, and ZmMOC1 regulate branching and growth. Rice’s architecture is shaped by OsMAX1, while in sugarcane, a suite of genes including SoMAX2, SoMAX3, SoMAX4-1, SoMAX4-2, and SoTB1 enhances tillering by modulating strigolactone pathways.

**Figure 1** shows the roles of specific regulatory genes in controlling plant architecture and tillering across four major crops: wheat, maize, rice, and sugarcane. Each panel displays a crop plant with areas significantly influenced by these genes highlighted, underscoring their impact on growth patterns and plant structure.

This intricate network of genes and their interactions underlines the complex genetic foundation of tiller development. Understanding these genetic mechanisms is essential for breeding programs aimed at developing crop varieties with optimized plant architecture, enhancing both yield and adaptability to various growing conditions. **Table 2** details various genes involved in tiller development across multiple plant species, highlighting their roles in axillary meristem formation, bud outgrowth regulation, and strigolactone signaling and biosynthesis. These genes collectively influence essential developmental processes through hormones such as auxins, gibberellins, cytokinins, and strigolactones. They are positively correlated with tiller number, spikelet development, grain number determination, and plant stress resistance. Through their interactions and regulatory mechanisms, they play critical roles in optimizing plant architecture and yield across rice, maize, wheat, sugarcane, and other species.

**Table 2:** Genes involved for tiller development.

Gene	Plant	Function	References
MOC1	Rice (*Oryza sativa*)	Axillary meristem formation	(Zhang et al., 2021)
MOC3/TAB1/SRT1	Rice (*Oryza sativa*)	Axillary meristem formation	(Shao et al., 2019)
LAX1	Rice (*Oryza sativa*)	Axillary meristem formation	(Oikawa and Kyozuka, 2009)
LAX2	Rice (*Oryza sativa*)	Spikelet and lemma development	(Dai et al., 2022)
FON1	Rice (*Oryza sativa*)	Stem cell maintenance	(Tanaka and Hirano, 2020)
SLR1	Rice (*Oryza sativa*)	Modulation of plant height and tiller number	(Liao et al., 2019)
TAD1	Rice (*Oryza sativa*)	Regulation of auxin	(Hussien et al., 2014)
OsTB1	Rice (Oryza sativa)	Tillering regulation	(Guo et al., 2013)
D3	Rice (Oryza sativa)	Strigolactone signaling	(Umehara et al., 2010)
D14	Rice (Oryza sativa)	Strigolactone receptor	(Luo et al., 2019)
MOC1 and MOC3	Rice (Oryza sativa)	Axillary meristem formation	(Shao et al., 2019)
OsMAX1	Rice (Oryza sativa)	Strigolactone biosynthesis	(Ito et al., 2022)
OsSPL14	Rice (Oryza sativa)	Tillering and yield regulation	(Li Y. et al., 2022)
OsMADS57, OsSPL2, OsSPL16, OsSPL17 and SPL18	Rice (Oryza sativa)	Tillering and plant architecture regulation	(Yan Y. et al. 2021)
D53	Rice (Oryza sativa)	Strigolactone signaling	(Soundappan et al., 2015)
D3, D10, D14, D27	Rice (*Oryza sativa*)	Strigolactone biosynthesis	(Wang Y. et al., 2020)
Os1900, and MAX1-like gene	Arabidopsis, Rice (*Oryza sativa*)	Strigolactone biosynthesis	(Cui et al., 2023)
HTD1	Rice (*Oryza sativa*)	Tillering and yield regulation	(Wang Y. et al., 2020)
TB1, OsTB1 *OsTb2*	Maize (Zea mays) and Rice (*Oryza*	Branch development	(Lyu et al., 2020)
tb1	Maize (Zea mays)	Inflorescence architecture	(Studer et al., 2011)
ramosa1	Maize (Zea mays)	Inflorescence architecture	(Strable et al., 2023)
ramosa2	Maize (Zea mays)	Axillary meristem formation	(Li Z. et al., 2022)
ZmMOC1	Maize (Zea mays)	Inflorescence development	(Yan et al., 2020)
ba1	Maize (Zea mays)	Tillering regulation	(Wang et al., 2022)
ZmTB1	Maize (Zea mays)	Strigolactone signaling	(Mansilla et al., 2023)
ZmD14	Maize (Zea mays)	Tillering regulation	(Fan et al., 2023)
Tin	Wheat (Triticum aestivum)	Strigolactone biosynthesis	(Kebrom et al., 2012)
TaD27-B	Wheat (Triticum aestivum)	Tillering regulation	(Zhao B. et al., 2020)
TaSPL14	Wheat (Triticum aestivum)	Strigolactone biosynthesis	(Gupta et al., 2023)
TaMAX1	Wheat (Triticum aestivum)	Tillering regulation	(Sigalas et al., 2023)
TaTB1	Wheat (Triticum aestivum)	Axillary meristem formation	(Zhao et al., 2018)
TaMOC1	Wheat (Triticum aestivum)	Flowering time and tillering regulation	(Yang et al., 2023; Zhang et al., 2015)
VRN1	Wheat (Triticum aestivum)	Flowering time and tillering regulation	(Chumanova et al., 2020)
FT1	Wheat (Triticum aestivum)	Regulation of tillering	(Gauley and Boden, 2021)
TN1	Bread Wheat	Strigolactone biosynthesis	(Dong et al., 2023)
HvMAX1	Barley (Hordeum vulgare)	Strigolactone biosynthesis	(Valivand, 2023; Wang et al., 2018)
HvTB1	Barley (Hordeum vulgare)	Tillering regulation	(Koppolu and Schnurbusch, 2019)
HvD14	Barley (Hordeum vulgare)	Strigolactone perception	(Zhou et al., 2020)
HvMAX1	Barley (Hordeum vulgare)	Strigolactone biosynthesis	(Valivand, 2023; Wang et al., 2018)
INTERMEDIUM-C	Barley (Hordeum vulgare)	Internode length regulation	(Ramsay et al., 2011)
HvSPL14	Barley (Hordeum vulgare)	Tillering regulation	(Thiel et al., 2021)
LSG1-2, ERF1-2, SHKA, TIL, HSP18.1, HSP24.1, HSP16.1, HSFA6A	Sugarcane (Saccharum spp.)		(Tang et al., 2023)
SoMAX2, SoMAX3, SoMAX4-1, SoMAX4-2 and SoTB1	Sugarcane (Saccharum spp.)	Strigolactone signaling	(Karno, 2007)https://espace.library.uq.edu.au/view/UQ:135076
T0, T1 and T2	Sugarcane (Saccharum spp.)	Key genes and pathways involved in tiller development	(Yan H. et al., 2021)
qPCTR-Y8-2, qRSR-R51 and qRSR-Y43-2	Sugarcane (Saccharum spp.)	Genetic improvement of tillering and ratooning traits	(Wang T. et al., 2023)
ScGAI	Sugarcane (Saccharum spp.)	Regulation of tillering and culm development	(Garcia Tavares et al., 2018)
GA3 and paclobutrazol	Sugarcane (Saccharum spp.)	Growth and development regulation	(Luo et al., 2023)

## 3 Genetic engineering approaches to enhance tiller development

The integration of multi-omics findings encompassing genomic, transcriptomic, proteomic, and metabolomic analyses has transformed agricultural sciences and crop breeding programs, providing deep insights into the genetic and molecular mechanisms underlying essential agronomic traits (Wang M. et al., 2023; Roychowdhury et al., 2023). This multi-omics knowledge is crucial for refining crop breeding techniques, enabling more precise identification of genetic markers associated with desirable traits, such as increased yield, disease resistance, and stress tolerance (Wang M. et al., 2023). One key application of multi-omics integration is in identifying markers linked to traits like tiller number, which can be leveraged in marker-assisted selection (MAS) and genomic selection (GS). While MAS is effective for traits governed by a few major genes, GS is more suitable for polygenic traits like tiller number, as it uses genome-wide markers to predict breeding values. For instance, major QTLs such as MOC1 and HTD1, associated with tiller number in rice, have been successfully incorporated into breeding programs through MAS (Takai, 2024). Meanwhile, GS models are increasingly applied in wheat and maize to improve tillering by integrating genomic prediction models that account for multiple small-effect loci. Combining these approaches with multi-omics data enhances the precision and efficiency of breeding strategies targeting optimal tiller development (Bunjkar et al., 2024).

This multi-omics approach also aids in understanding the genetic control of complex traits, such as tillering, under varying environmental conditions. This knowledge is instrumental in identifying candidate genes for genetic modification, allowing breeders to create cultivars adapted to specific environmental contexts (Yan et al., 2020). In some instances, transgressive segregation for tiller number has been observed, where progeny exhibit a greater number of tillers than either parent, due to the recombination of complementary alleles. This phenomenon has been noted in rice and wheat, demonstrating its potential for achieving superior tillering traits in breeding programs (Koide et al., 2019). In addition, genomic selection and hybrid breeding methods are being optimized through multi-omics insights, particularly in understanding the molecular basis of heterosis (hybrid vigor) to create superior hybrids that maximize yield and other beneficial traits (Haile Gidamo, 2023; Budhlakoti et al., 2022). This customization allows breeding strategies to adapt to specific environmental conditions, enhancing resilience and adaptability. Moreover, multi-omics data support precise modifications through advanced gene-editing techniques like CRISPR-Cas9, which enable specific genome alterations, further advancing the development of crops with improved tillering traits.

Genetic engineering (GE) has shown considerable promise in modulating tiller number, though research in this area is ongoing. GE techniques, such as CRISPR-Cas9, have been used to target key genes involved in tillering (Gan and Ling, 2022). For example, in rice, the CRISPR-Cas9-mediated knockout of the MONOCULM 1 (MOC1) gene, essential for tiller initiation, has been shown to enhance tillering. Similarly, genes involved in strigolactone biosynthesis and signaling, such as HTD1 and D14, have been modified to influence tiller development (Takai, 2024). Although promising, the success of GE in controlling tiller number is variable due to the complex interplay of genetic and environmental factors that regulate tillering. This variability underscores the need for a comprehensive understanding of the genetic networks and environmental interactions that govern tiller development (Reynolds et al., 2020). For example, modifying genes such as TaSPL14 and TaMOC1 in wheat has demonstrated potential in influencing tiller number. However, achieving consistent results across diverse environments remains challenging. In maize, targeting genes like ZmTB1 and ZmD14 has shown similar promise, though the polygenic nature of tillering complicates the outcomes (Yang et al., 2023; Zhang et al., 2015; Mansilla et al., 2023; Fan et al., 2023). The responsiveness of tiller number to genetic engineering emphasizes the need for further research focused on fine-tuning gene-editing techniques, understanding gene-environment interactions, and integrating multi-omics data to develop robust strategies for enhancing tiller development.

Overall, studies highlight the potential of genetic engineering in controlling tiller numbers, though additional research is necessary to refine these approaches. Integrating GE with traditional breeding techniques and incorporating insights into genetic and environmental factors that influence tillering will be essential for future success. This multidisciplinary approach holds promise for developing resilient, high-yield crop varieties capable of sustaining tiller development under diverse environmental conditions.

## 4 Quantitative trait loci mapping in tiller development

Quantitative traits, such as tillering in crop plants, display continuous variation due to the combined influence of multiple genes, known as polygenes. Unlike simple traits controlled by single genes, these quantitative traits exhibit a spectrum of phenotypic expressions, highlighting their complex genetic foundations (Bhatia, 2020). Tiller development, marked by variation in the number and vigor of tillers among plants, exemplifies such a trait, driven by the cumulative effects of numerous genes (Yan et al., 2023). Advances in QTL mapping have deepened our understanding of the genetic regulation of tillering across major crops, pinpointing specific loci associated with traits like tiller number, angle, and strength. In rice (*Oryza sativa*), key QTLs such as *qTN1* and *qTAC1* on chromosomes 1 and 4 have been identified, with *qTN1* influencing tiller number and *qTAC1* regulating tiller angle critical for optimizing plant architecture and light interception (Waite et al., 2018). In wheat (*Triticum aestivum*), the QTL *QTn.cau-2A* has been linked to increased tiller numbers and is associated with enhanced grain yield, making it a valuable target for breeding high-yielding wheat varieties (Wang et al., 2024).

In maize (*Zea mays*), QTL mapping has uncovered loci such as *qTb1* on chromosome 1, which influences tillering through its regulation of the *TEOSINTE BRANCHED1* (TB1) gene a primary determinant of the single-stalk growth habit in maize (Studer et al., 2011). Even in polyploid crops like sugarcane (*Saccharum* spp.), with its intricate genome, QTL mapping has identified loci such as *qTC-SC1.1* on chromosome 1, which is linked to tiller count and biomass production—traits essential for bioenergy production and yield optimization (Ming et al., 2001).

These QTL mapping advancements not only highlight key loci but also underscore the polygenic nature of tillering traits, where numerous QTLs with small to moderate effects collectively shape tillering. Integrating QTL mapping with other omics approaches, such as transcriptomics, has allowed researchers to identify candidate genes within these QTL regions, thus advancing the molecular understanding of tiller development. QTL mapping is a sophisticated technique for pinpointing specific genomic regions, or loci, that harbor genes influencing complex traits. By examining the association between genetic markers specific DNA sequences and trait variation across populations, researchers can determine the number, locations, and effects of loci involved in traits like tillering. This method is fundamental for elucidating how multiple genes collectively impact complex traits, enabling targeted breeding strategies (Aswini et al., 2023).

A crucial component of QTL mapping is linkage analysis, which assesses the relationship between genetic markers and traits. By identifying markers that are consistently co-inherited with a trait, researchers can infer their proximity to relevant genes. Linkage analysis monitors the co-segregation of markers with traits, indicating physical closeness on the chromosome and aiding in the localization of genes influencing the trait (Ibrahim et al., 2020; Daware et al., 2020). The construction of specific mapping populations is essential for QTL studies. These populations, derived from crossing two parent plants with contrasting characteristics in the trait of interest, exhibit a wide range of genetic diversity crucial for QTL mapping accuracy. For example, *F2* populations, created by selfing or intercrossing *F1* individuals, provide broad genetic combinations for initial QTL identification. Backcross populations refine these findings by isolating the effects of individual QTLs within a relatively uniform genetic background, allowing more precise estimations of QTL impacts (Nam et al., 2021; Shang et al., 2021). For tiller development, both *F2* and backcross populations offer valuable insights into genetic factors influencing traits like tiller number, angle, and vigor. These insights are critical for plant breeders aiming to develop crop varieties optimized for specific tillering attributes, ultimately enhancing crop yield and productivity (Zhao et al., 2022).

In summary, QTL mapping, particularly when combined with linkage analysis and diverse mapping populations, provides a robust foundation for dissecting complex traits like tiller development. This approach not only elucidates the genetic architecture of these traits but also supports breeding programs focused on improving crop characteristics crucial for agricultural productivity.

### 4.1 QTL mapping in major crops (e.g., sugarcane, rice, wheat and maize)

QTL mapping has been crucial in identifying genetic regions associated with tillering traits in major crops, providing insights that support breeding for enhanced yield and adaptability.

**Figure 2:**
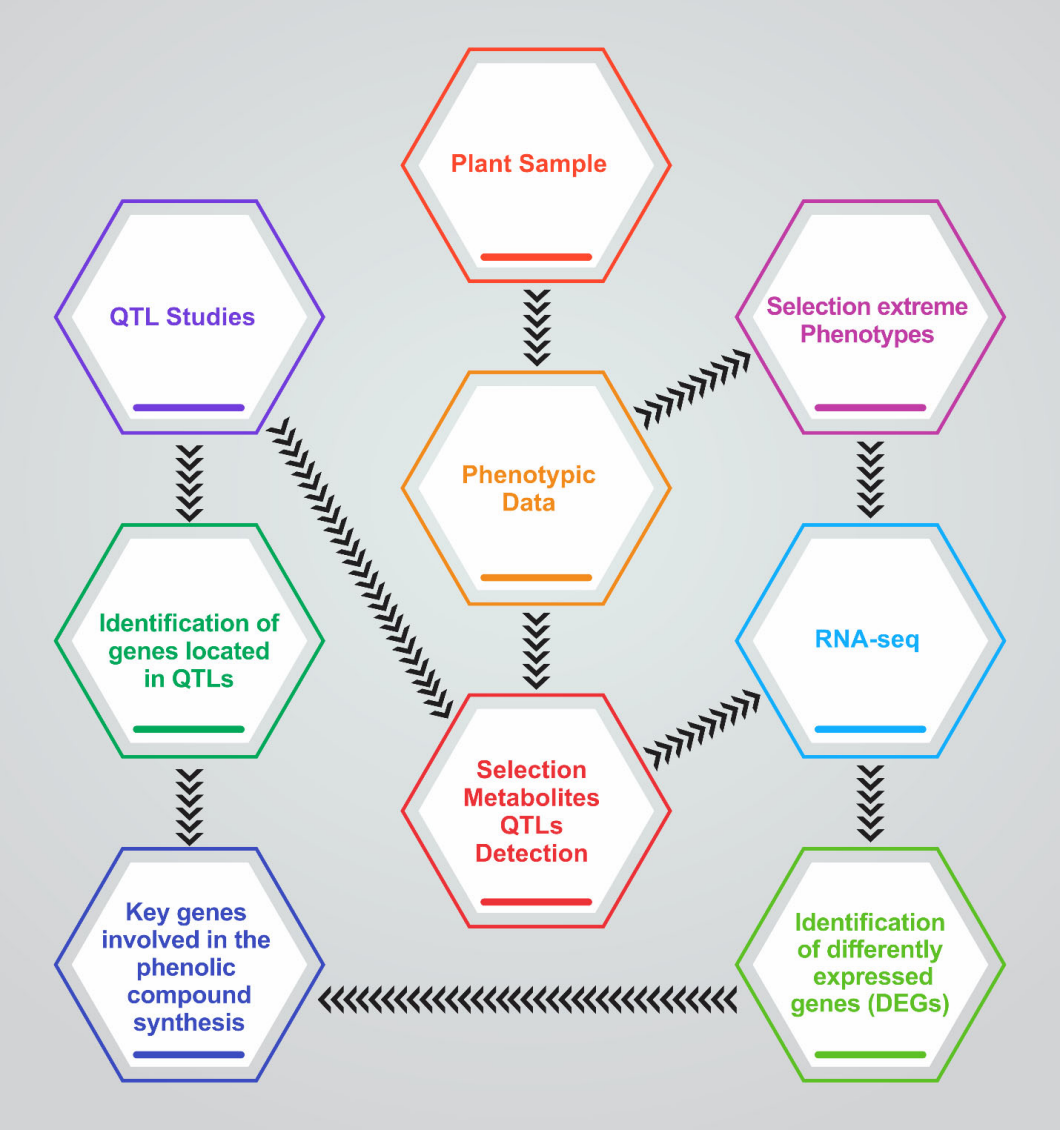
Roadmap diagram illustrating the integrative approach to identifying key genetic components in plant samples. The process begins with plant sample collection, followed by phenotypic data analysis and selection of extreme phenotypes. QTL studies and RNA-seq analysis are conducted in parallel to pinpoint genes located within QTLs and differentially expressed genes (DEGs), respectively. Central to the workflow is the selection of metabolites QTLs detection, which links phenotypic traits to molecular findings. The culmination of this process is the identification of key genes involved in phenolic compound synthesis, providing insights into the genetic basis of plant traits.

In sugarcane, a complex polyploid crop, QTL mapping has linked loci to tiller number and size, directly impacting biomass production. Studies by Ming et al. identified QTLs associated with stalk count, essential for yield and bioenergy production (Ming et al., 2001; Wang T. et al., 2023). In the context of these studies, *Phenotypic Data* would include measurements of tiller number, size, angle, and strength across crop species, forming the basis for detecting *Selection Metabolite QTLs* metabolites that correlate with tillering traits. For example, in sugarcane, Ming et al.’s work demonstrates how genetic regions are linked to traits like stalk number, similar to identifying genes located in QTLs, as shown in **Figure 2**.

### 4.2 Relationship between phenolic compounds and tiller development

Phenolic compounds, such as flavonoids, lignins, and phenolic acids, play crucial roles in plant development, particularly in regulating tiller initiation, bud outgrowth, and stress responses. These secondary metabolites are involved in modulating hormone signaling pathways, including auxins, cytokinins, and strigolactones, which are key regulators of tillering.

1. **Hormonal Regulation:** Some phenolic compounds act as modulators of plant hormone activity. For example, flavonoids have been shown to influence auxin transport, affecting bud dormancy and tiller formation. In rice, reduced flavonoid accumulation leads to excessive tillering due to altered auxin distribution, disrupting the hormonal balance between apical dominance and lateral bud growth.
2. **Stress Response and Tiller Growth:** Phenolic compounds contribute to plant defense mechanisms against abiotic stresses such as drought and high temperatures, which directly impact tillering. Accumulation of lignin, a major phenolic polymer, strengthens cell walls and enhances plant resilience, indirectly supporting the persistence and growth of tillers under stressful conditions.
3. **Genetic Associations:** Recent QTL mapping studies have identified co-localization of genes involved in phenolic biosynthesis and tillering traits. For example, genes regulating lignin biosynthesis (e.g., *PAL* and *C4H*) have been mapped near QTLs controlling tiller number in wheat and sugarcane, suggesting a genetic link between secondary metabolism and tiller development.

Understanding the role of phenolic compounds in tillering could offer novel breeding strategies, where selection for specific metabolite profiles may enhance tiller persistence and crop productivity. Further transcriptomic studies could help clarify the regulatory interactions between phenolic metabolism and tiller growth, leading to improved crop varieties with optimized tillering under diverse environmental conditions.

For rice, QTL mapping has identified loci such as *MONOCULM 1* (MOC1), critical for tiller initiation, providing valuable targets for improving plant architecture and yield (Shao et al., 2019; Xu et al., 2012). In wheat, QTLs affecting both the number and lifespan of tillers have been mapped, showing potential for breeding programs focused on yield optimization. Studies by Kebrom et al. highlighted loci related to tiller number and longevity, which are vital for maximizing productivity (Kuchel et al., 2007). Maize has benefited from QTL mapping for tillering traits, aiding in the development of varieties suitable for diverse planting densities and environmental conditions. Identified QTLs provide targets for breeding resilient, high-yield maize (Wang et al., 2023a; Zhang et al., 2022, 2014).

Through QTL mapping, key loci for tillering traits have been identified across these crops, supporting advanced breeding strategies such as marker-assisted selection and genetic engineering. This approach lays the foundation for developing high-yield, climate-adapted varieties, essential for sustainable agriculture.

## 5 Association studies in tiller development

Genome-Wide Association Studies (GWAS) have become invaluable in plant genetics, particularly for analyzing complex traits like tiller development (Uprety, 2023). Unlike traditional QTL mapping, which depends on controlled crossbreeding, GWAS leverages the extensive genetic variation found in natural populations to identify associations between genetic variants and traits (Xiao et al., 2022). Through GWAS, researchers have pinpointed key genetic loci linked to tillering traits across multiple crop species, thereby offering significant insights for crop improvement. In rice (*Oryza sativa*), GWAS has identified loci such as *OsSPL14*, which regulates both tiller number and plant height by modulating hormone levels, particularly strigolactones. These hormones inhibit axillary bud outgrowth, making *OsSPL14* a critical locus for breeding rice varieties with optimal tillering and plant architecture (Wang et al., 2015). In wheat (*Triticum aestivum*), the locus *TraesCS7B02G282100* on chromosome 7B, identified through GWAS, is associated with an increased tiller number and robustness, which enhances yield potential under diverse environmental conditions (Kebrom et al., 2012).

Barley (*Hordeum vulgare*) has also been a focus in tillering studies. GWAS analysis has identified the *HvSPL14* gene as a significant contributor to tiller development, enabling selective breeding for desired tillering traits tailored to specific environmental contexts (Valivand, 2023). In sorghum (*Sorghum bicolor*), GWAS has uncovered loci linked to drought tolerance that simultaneously impact tillering, illustrating the complex relationship between environmental stress adaptation and tiller number (Zhou et al., 2022). These findings highlight the importance of GWAS in uncovering genetic markers for tillering traits influenced by environmental conditions. Using these markers in marker-assisted selection (MAS) programs accelerates the breeding process, allowing breeders to develop crop varieties with optimized tillering suited to diverse growing environments and agricultural practices. Furthermore, integrating GWAS findings with transcriptomic data provides insights into the regulatory networks governing tillering, deepening our understanding of the gene-environment interactions central to this complex trait.

Genome-Wide Association Studies (GWAS) have significantly advanced plant genetics, particularly in analyzing complex traits like tiller development. These studies leverage the extensive genetic variation within natural populations, contrasting with traditional QTL mapping’s reliance on controlled cross-breeding (Xiao et al., 2022). Through GWAS, key genetic variants associated with tillering traits have been identified across different crops. In rice, GWAS has uncovered loci such as OsSPL14, which influences both tiller number and plant height by modulating hormone levels, particularly strigolactones, which inhibit axillary bud outgrowth (Wang et al., 2015). This discovery has significant implications for breeding rice varieties with optimal tillering and plant architecture. In wheat, the GWAS-identified locus TraesCS7B02G282100 on chromosome 7B is associated with increased tiller number and robustness, enhancing yield potential under varying environmental conditions (Kebrom et al., 2012). Barley (*Hordeum vulgare*) has been another focal point of tillering studies. GWAS analysis identified the *HvSPL14* gene as a major player in controlling tiller development, allowing for selective breeding of barley with desired tillering traits for different environmental contexts (Valivand, 2023). GWAS studies in sorghum (*Sorghum bicolor*) have identified loci linked to drought tolerance that simultaneously affect tillering, highlighting the complex relationship between environmental stress adaptation and tiller number (Zhou et al., 2022).

These findings underscore the importance of GWAS in identifying genetic markers for tillering traits that vary according to environmental conditions. By using these markers in marker-assisted selection (MAS) programs, breeders can expedite the development of crop varieties with optimized tillering, tailored to meet the demands of different growing environments and agricultural practices. Moreover, integrating GWAS findings with transcriptomic data enables the identification of regulatory networks governing tillering, enhancing our understanding of the gene-environment interactions involved in this complex trait.

### 5.1 Examples of successful association studies in different crops

Genome-Wide Association Studies (GWAS) have significantly advanced our understanding of tiller development across various crops, each yielding unique insights with practical implications for crop breeding. In rice (*Oryza sativa*), where tillering is a critical determinant of yield, GWAS has identified multiple loci associated with tiller number and density. These discoveries have facilitated the breeding of rice varieties with desirable tillering traits, directly enhancing grain yield (Ren et al., 2021; Sachdeva et al., 2024). For wheat (*Triticum aestivum*), GWAS has uncovered genetic markers associated with tiller number, among other yield-related traits (Khan et al., 2022; Mulugeta et al., 2023a; Pang et al., 2020; Schoen et al., 2023). Notable loci, including *qTN-7B.1* on chromosome 7B, provide valuable targets for breeding high-yielding cultivars. Candidate genes, such as *TraesKN5D01HG00080* linked to *qMtn-KJ-5D.1* and *TraesCS7B02G282100* for *qTN-7B.1*, offer potential molecular targets for improving wheat yield through tiller trait optimization (Mulugeta et al., 2023a).

Barley (*Hordeum vulgare*) has also been extensively studied using GWAS to understand traits related to plant architecture, crop management, and stress adaptation (Genievskaya et al., 2023). These studies have identified loci associated with grain quality traits, such as protein, starch, and lipid content, and have revealed marker-trait associations that improve yield under different environmental conditions, particularly drought (Genievskaya et al., 2022; Elbasyoni et al., 2022). These findings underscore the role of genetic markers in breeding barley varieties with improved resilience and quality traits. In maize (*Zea mays*), typically characterized by limited tillering, GWAS has contributed insights into the genetic basis of tiller development, informing breeding strategies for plant structure optimization. Understanding maize’s unique evolutionary and tillering background provides a foundation for breeding programs aimed at maximizing productivity and suitability for varied environments (Wu X. et al., 2023). Sorghum (*Sorghum bicolor*) has benefited from GWAS identification of loci associated with tiller number and size, traits crucial for both grain yield and forage quality. This genetic information supports breeding sorghum varieties suited to diverse agricultural and environmental contexts, enhancing adaptability and yield (Lee et al., 2023).

Finally, sugarcane (*Saccharum* spp.), known for its polyploid genome and value in bioenergy, has been studied using GWAS to reveal the genetic factors behind tiller number and stalk size. These discoveries are pivotal for breeding sugarcane varieties with improved biomass productivity, essential for bioenergy efficiency and industry sustainability (Barreto et al., 2019). These examples from diverse crops illustrate the power of GWAS in elucidating the complex genetics of tiller development. The genetic insights from these studies serve as invaluable resources for developing crop varieties with optimized tillering traits, enabling breeders to meet the specific demands of varied agricultural environments and practices, ultimately contributing to higher yields, enhanced adaptability, and improved crop quality.

## 6 Transcriptome analysis in understanding tiller development

Transcriptome analysis has become an indispensable technique in modern molecular biology, especially in understanding complex physiological processes like tiller development in plants. This approach involves studying the transcriptome, which encompasses the entire range of RNA transcripts produced by the genome under specific conditions. The most prominent technique in this field is RNA Sequencing (RNA-Seq). RNA-Seq allows for comprehensive sequencing of all RNA molecules within a sample, including mRNA, non-coding RNA, and small RNA (Upton et al., 2023). It provides a detailed view of the transcriptome, offering insights into gene expression levels, splicing variations, and the discovery of new transcripts. Before the advent of RNA-Seq, microarrays were the standard tool for transcriptome analysis. This method involves hybridizing cDNA to an array of complementary DNA probes on a chip and is effective for comparing gene expression across different samples. However, microarrays have limitations, such as being restricted to detecting transcripts that match the array’s probes (Tyagi et al., 2022).

Quantitative Real-Time PCR (qRT-PCR) is another crucial technique often used alongside RNA-Seq. qRT-PCR is highly sensitive and precise for quantifying specific RNA transcripts, making it ideal for validating findings from RNA-Seq and microarrays. The transcriptome analysis generates vast amounts of data, necessitating advanced bioinformatics for data interpretation. This includes aligning sequencing reads to reference genomes, quantifying gene expression, and identifying differentially expressed genes, as well as employing computational methods to decipher gene regulatory networks (Li et al., 2022; Zhao et al., 2021). In the study of tiller development, transcriptome analysis has been key to identifying genes and pathways involved in this complex trait. By comparing the transcriptomes of plants with varying tillering characteristics, researchers can pinpoint differentially expressed genes that are likely involved in controlling tiller growth. This information is essential for understanding the molecular mechanisms behind tiller development and for developing strategies to manipulate this trait for crop improvement.

Overall, transcriptome analysis, with its comprehensive and dynamic view of gene expression, offers invaluable insights into the regulatory networks that govern important plant traits like tiller development. This technique is a cornerstone in plant biology, facilitating advanced research and development in crop breeding and agricultural practices.

### 6.1 Role of gene expression profiling in tiller development

Gene expression profiling is a crucial tool for dissecting the molecular mechanisms underlying tiller development in plants. By analyzing patterns and levels of gene expression, researchers can identify key genes involved in the initiation and maturation of tillers, shedding light on the regulatory processes that govern this important trait. This approach helps identify differentially expressed genes in plants with varying tillering capacities, offering insights into the genes’ roles in tiller growth and development (Martínez-Iglesias et al., 2023).

In rice (*Oryza sativa*), for instance, the *MONOCULM 1 (MOC1)* gene plays a pivotal role in tiller bud initiation. *MOC1* promotes axillary meristem formation, which leads to lateral tiller production. Mutations in *MOC1* produce rice plants with a single stem and no tillers, underscoring its critical role in branching and tiller formation. Studies have shown that *MOC1* expression peaks during early axillary bud outgrowth, linking its activity directly to tiller number (Li et al., 2003).

In maize (*Zea mays*), the *TEOSINTE BRANCHED1 (TB1)* gene acts as a negative regulator of tillering by suppressing lateral shoot growth. High expression of *TB1* in maize, which has a single dominant stalk, contrasts with its reduced expression in teosinte, the wild ancestor of maize, allowing multiple tillers to develop. This gene’s expression responds to environmental cues like light and nutrients, influencing plant architecture by concentrating resources in the main shoot for optimized grain production (Doebley et al., 1997). Similarly, in wheat (*Triticum aestivum*), the *TaSPL14* gene, a homolog of rice *SPL14*, regulates tiller number through its interaction with strigolactones. Overexpression of *TaSPL14* results in reduced tillering due to increased strigolactone biosynthesis, which inhibits bud outgrowth. Reduced expression of *TaSPL14* leads to increased tillering by limiting strigolactone levels, allowing more axillary buds to develop into tillers (Gupta et al., 2023). This highlights how expression levels of regulatory genes like *TaSPL14* can modulate hormonal pathways crucial for tiller development. *OsSPL14* in rice provides another example, as it influences both tiller number and plant height through interactions with miR156, a microRNA known to regulate developmental timing. Upregulated *OsSPL14* expression results in fewer tillers but a stronger central stem, enhancing the plant’s capacity to support higher grain yield (Wang et al., 2015). This gene’s role exemplifies the complex balance between tiller development and overall plant productivity.

Gene expression profiling thus enables the identification of key regulatory genes involved in tillering and helps clarify how expression levels influence tiller development. Profiling these genes under different environmental and developmental conditions reveals critical insights into the molecular mechanisms that govern plant architecture and yield potential. Tiller growth is regulated by complex networks involving hormone signaling pathways, particularly cytokinins and strigolactones, which are central to plant development (Wu F. et al., 2023; Yan et al., 2023). Gene expression profiling helps elucidate the molecular pathways that control tillering and how these pathways respond to internal and external factors. In rice, studies have shown that genetic factors, endogenous hormones, and environmental conditions are key regulators of tillering. In wheat, circular RNAs (circRNAs) are involved in tiller formation and development, while the *TFL1* gene regulates tiller number through interactions with auxin and cytokinin signaling pathways (Sun et al., 2023). Such findings demonstrate how gene expression profiling can reveal the complex mechanisms underlying tiller development. Environmental factors, such as light, nutrient availability, and water, also play a major role in influencing tiller-related gene expression. Gene expression profiling can thus provide insights into how these factors impact tillering, revealing the plant’s adaptive responses to different conditions (Li et al., 2022). This information is invaluable for crop breeding as it enables the selection or genetic modification of plants for optimal tillering traits, ultimately maximizing yield potential (Yadesa, 2022).

Additionally, gene expression profiling aids in identifying novel genes involved in tiller development, which can be further characterized in functional genomics studies. These findings broaden our understanding of plant growth and facilitate the development of crop varieties with enhanced performance and adaptability. Overall, gene expression profiling offers a comprehensive view of the dynamic gene regulatory events that drive tiller development, advancing our knowledge of plant biology and supporting targeted crop improvement strategies.

## 7 Integration of multi-omics approaches for tiller development

The integration of multi-omics approaches, including Quantitative Trait Loci (QTL) mapping, association analysis (such as Genome-Wide Association Studies), and transcriptomics, offers a comprehensive strategy for elucidating tiller development in plants. This holistic methodology leverages the strengths of each technique to unravel the complex genetic and molecular underpinnings of tiller development (Shariatipour et al., 2021). By combining QTL mapping and GWAS, researchers can identify critical genomic regions associated with tillering traits. However, understanding the functional aspects of these regions requires the additional layer of transcriptomics, which elucidates gene expression dynamics and regulatory (Bilgrami et al., 2020).

This synergistic approach enhances gene discovery and the functional annotation of identified genomic regions. Correlating transcriptomic data with genetic markers from QTL and GWAS enables researchers to pinpoint candidate genes that are crucial for variations in tiller development. This is especially valuable for genomic regions where functions are not previously known or are poorly understood. Furthermore, integrating these omics techniques sheds light on the complex genetic networks that govern tillering. It helps unravel the interplay and contribution of different genes, including the identification of key regulatory elements that control the expression of genes involved in tiller growth and development (Shao et al., 2022).

From a practical perspective, the integration of these multi-omics approaches holds significant implications for plant breeding and genetic engineering. A more comprehensive understanding of the genetic factors that influence tiller development allows breeders to make informed selections for desirable traits (Kinose et al., 2023). Similarly, genetic engineers can target specific genes or pathways identified through this integrated framework to develop crop varieties with optimized tillering characteristics, which is crucial for improving crop yield and adaptability (Shao et al., 2022).The combined use of QTL mapping, association analysis, and transcriptomics provides a robust framework for studying tiller development. This multi-omics approach not only deepens our understanding of plant biology but also equips breeders and geneticists with the knowledge and tools necessary for developing improved crop varieties through targeted breeding and genetic modification strategies.

### 7.1 Integrating transcriptomics with QTL and association studies

Integrating transcriptomics with Quantitative Trait Loci (QTL) mapping and Genome-Wide Association Studies (GWAS) provides a powerful approach to decipher the genetic basis of tiller development (Zhu et al., 2023). This integration links specific genetic regions to functional gene expression, uncovering molecular mechanisms underlying this complex trait. While transcriptomics reveals active gene expression patterns, QTL mapping and GWAS identify loci associated with traits like tillering. By combining these methods, researchers can correlate the expression of certain genes with loci identified by QTL and GWAS, enhancing our understanding of how these genes influence tiller development (Li et al., 2023).

One challenge in QTL and GWAS is functionally annotating loci, particularly when they do not align directly with known genes. Transcriptome analysis addresses this by providing gene expression data in these regions, supporting hypotheses about their roles in tiller development. This integrated approach helps pinpoint candidate genes within QTL regions or near GWAS markers with expression patterns related to tillering, making them prime candidates for functional analysis (Shariatipour et al., 2021). Additionally, this method offers insight into gene networks and pathways involved in tillering, revealing key regulatory genes that coordinate the expression of other genes associated with tiller development. This comprehensive view is invaluable for crop breeding, as understanding genetic and expression profiles linked to favorable tillering traits enables more informed selections to improve crop yield and resilience. Integrating transcriptomics with QTL and GWAS thus bridges the gap between genetic variation and functional roles in plants, refining breeding programs and genetic engineering efforts for enhanced productivity and adaptability.

### 7.2 Bridging omics technologies: innovations and future opportunities

Multi-omics integration in studies of complex traits, such as tiller development, presents challenges, including data complexity, functional annotation, and resource allocation. The vast data from genomic, transcriptomic, and phenotypic analyses require substantial computational resources and bioinformatics expertise for management, processing, and interpretation (de Maturana et al., 2019; Palsson and Zengler, 2010). Advanced tools are necessary to perform these integrative analyses, highlighting the importance of interdisciplinary collaboration, which demands expertise across genetics, molecular biology, bioinformatics, and plant physiology. Effective communication and alignment of goals across domains are critical for project success, though equitable resource sharing and training in interdisciplinary methods remain challenging (Baxter, 2023).

Functional annotation is particularly challenging in non-model crops with less-characterized genomes. Identifying loci and expression patterns related to traits like tillering often requires additional experimentation to determine gene functions, which is complicated by factors such as gene duplication and epigenetic modifications (Yang et al., 2020). High costs and limited access to advanced omics technologies especially in developing countries further hinder research, limiting some institutions’ capacity to conduct multi-omics studies due to expenses associated with sequencing, data management, and computational infrastructure. This financial and knowledge gap may also lead to disparities in scientific contributions globally.

Addressing these challenges is essential for advancing multi-omics studies in plant biology. Solutions include continued development of bioinformatics tools, enhanced computational capabilities, fostering interdisciplinary collaborations, and global initiatives supporting research in developing countries. Such efforts will enable the full potential of multi-omics, advancing our understanding of complex biological systems and contributing to crop improvement.

The future of multi-omics integration, particularly for traits like tiller development, promises significant advances. Bioinformatics and data analysis are set to benefit from new tools for efficient data storage, retrieval, and preprocessing, with user-friendly interfaces democratizing access to advanced bioinformatics. Incorporating advanced statistical methods, machine learning, and artificial intelligence will address challenges related to high data dimensionality, revealing new insights critical for crop improvement (O’Connor et al., 2023).

In precision breeding, multi-omics data offer detailed genetic and molecular maps, allowing breeders to focus on specific genes or pathways for targeted improvement. This data complements gene-editing technologies like CRISPR-Cas9, which enable precise genomic modifications to enhance traits, accelerating the development of optimized crop varieties (Farinati et al., 2023). Integrating multi-omics insights with gene-editing offers precision in crop improvement, targeting genes related to tiller development to optimize yield and robustness in diverse environments, effectively addressing genetic complexity and limited diversity (Banakar, 2023; Kocsisova and Coneva, 2023).

A systems biology approach, combining multi-omics data with gene editing, holds promise for creating crop varieties optimized for tillering and yield. This strategy customizes crops to specific environments and goes beyond traditional breeding limitations, advancing the improvement of complex traits (Kumar et al., 2021). Expanding multi-omics research to non-model crops is vital for global food security, providing insights that adapt agricultural practices to local conditions and enhancing resilience. Collaborative efforts and resource-sharing will be essential for advancing research in this area, benefitting both local communities and global agriculture (Jaramillo-Botero et al., 2022; Yaqoob et al., 2023).

**Figure 3:**
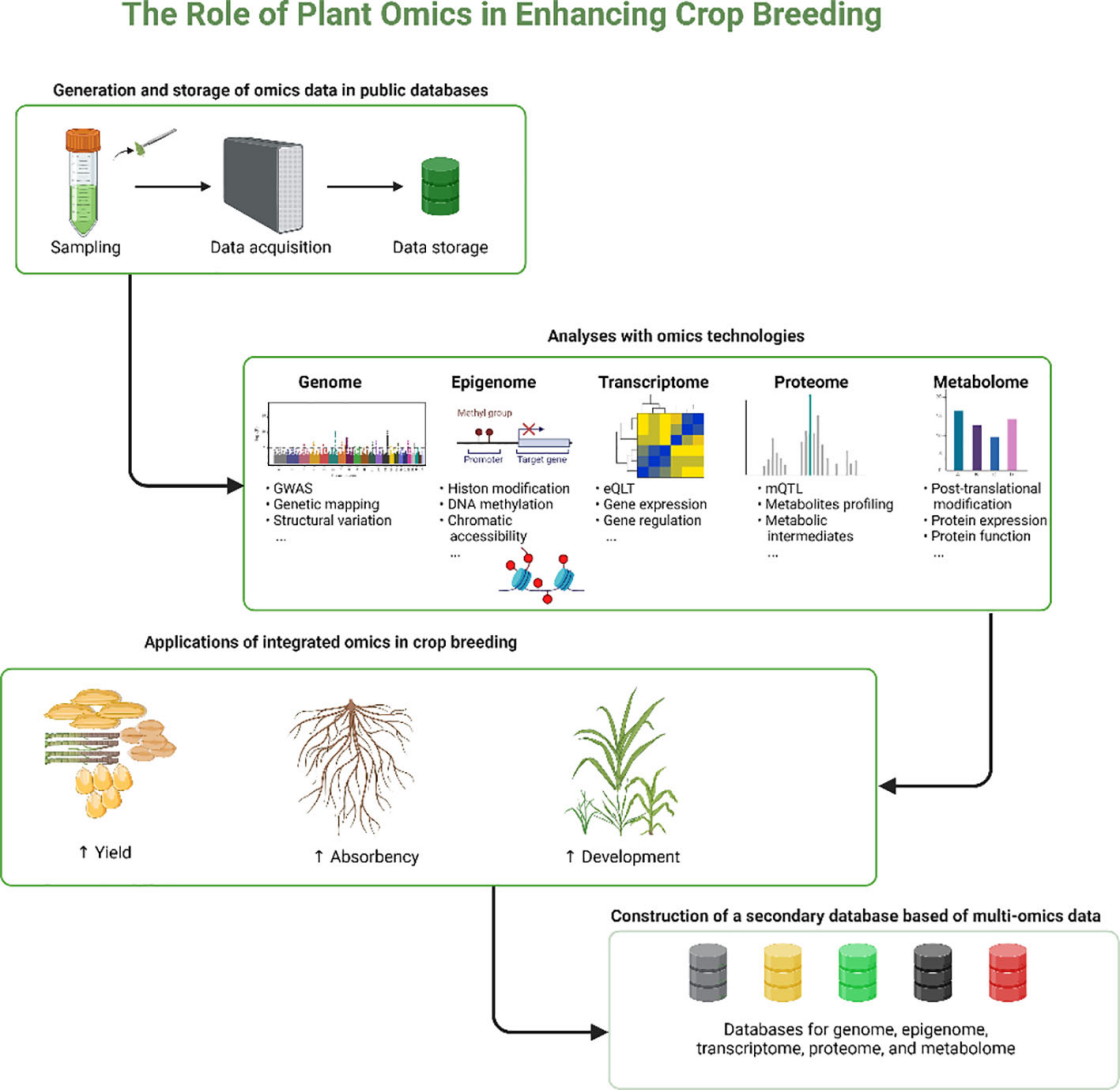
The diagram shows the systematic process of enhancing crop breeding using integrated omics technologies. Starting with the collection and storage of data, it progresses through detailed analyses across multiple omics fields genomic, epigenomic, transcriptomic, proteomic, and metabolomic. Each field contributes to a comprehensive understanding of plant biological processes, which is applied to improve crop yield, nutrient absorption, and overall development. These integrated insights lead to the creation of a secondary, multi-omics database, enhancing the capabilities and efficiency of crop breeding programs.

The role of plant omics in enhancing crop breeding, provides a detailed visual representation of how various omics technologies are utilized to advance crop breeding (**Figure 3**). Overall, the integration of multi-omics approaches in plant biology, especially for complex traits like tiller development, presents promising avenues for significant advancements in bioinformatics, precision breeding, gene editing, and the study of non-model crops. These developments are expected to transform our understanding of plant biology, driving innovations in crop breeding and agricultural productivity.

## 8 Conclusion and future perspectives

In conclusion, the exploration of tiller development through a multi-faceted approach encompassing QTL mapping, association studies, and transcriptome analysis has significantly advanced our understanding of the genetic and molecular foundations essential for improving crop yields. This comprehensive review underscores the pivotal role of tiller development in enhancing agricultural productivity and addressing the global challenge of food security. The integration of multi-omics techniques has not only illuminated the complex genetic and environmental interactions influencing tiller development but also paved the way for the development of crop varieties with optimized tillering traits. The findings highlight the critical need for future research to incorporate a multidisciplinary perspective, blending insights from genetics, molecular biology, and environmental science to foster resilient and high-yielding crops. Specifically, the study of key genes such as *TaMAX1, TaMOC1, and TN1 in wheat, ZmTB1, ZmD14, and ZmMOC1* in maize, and *MAX1*-like genes in rice, as well as regulatory genes in sugarcane like *SoMAX2, SoMAX3*, *SoMAX4-1, SoMAX4-2*, and *SoTB1*, showcases the potential for targeted genetic improvements. These insights not only highlight pivotal genetic loci and gene expression patterns essential for optimized tiller development but also pave the way for the development of new crop varieties through precision breeding and genetic engineering. Ultimately, this will contribute significantly to enhanced agricultural productivity and global food security.

The future of agricultural research is increasingly defined by the integration of advanced technologies across various scientific domains, emphasizing the need for collaborative and multidisciplinary approaches. Emerging technologies like single-cell sequencing, CRISPR-Cas systems, and advanced bioinformatics, including machine learning and artificial intelligence, are at the forefront, offering new insights into plant biology at an unprecedented level of detail. These technologies enable a deeper understanding of genetic, transcriptomic, and epigenomic mechanisms, facilitating targeted genetic modifications and a comprehensive view of cellular processes, gene regulation, and stress responses in plants. The integration of genomics, transcriptomics, metabolomics, and proteomics promises a holistic approach to understanding plant biology, aiding in unraveling complex networks of gene regulation, protein function, and metabolic pathways. This comprehensive view is crucial for advancing crop improvement strategies, enhancing traits such as yield, disease resistance, and environmental stress tolerance. The significance of phenomics and high-throughput phenotyping technologies is also growing, allowing for the rapid measurement of plant traits to correlate genomic data with real-world performance under varying conditions.

Climate change poses significant challenges to agricultural research, particularly in areas like tiller development, which is vital for crop productivity. Research must now focus on understanding plant responses to abiotic stresses exacerbated by climate change, such as drought, heat, and salinity, and their impact on tillering patterns and plant health. Advanced modeling techniques and predictive analytics will be essential in simulating climate change scenarios and guiding the breeding of resilient crop varieties. An interdisciplinary approach, integrating plant science, climatology, and agricultural economics, is key to addressing the complexities introduced by climate change and developing sustainable solutions. Collaborative efforts, spanning cross-institutional partnerships and public-private collaborations, are critical for pooling resources and expertise. Global research networks and engagement with farming communities are essential for the dissemination and application of knowledge across different agricultural contexts. Furthermore, interdisciplinary training and education programs are vital for equipping researchers with the necessary skills for effective collaboration and innovation.

In summary, the convergence of emerging technologies and collaborative, interdisciplinary approaches mark a new era in agricultural research. These efforts are geared towards deepening our understanding of plant biology, addressing the challenges posed by climate change, and contributing to sustainable agricultural practices and global food security.
